# SNAPS: Sensor Analytics Point Solutions for Detection and Decision Support Systems

**DOI:** 10.3390/s19224935

**Published:** 2019-11-13

**Authors:** Eric S. McLamore, Shoumen Palit Austin Datta, Victoria Morgan, Nicholas Cavallaro, Greg Kiker, Daniel M. Jenkins, Yue Rong, Carmen Gomes, Jonathan Claussen, Diana Vanegas, Evangelyn C. Alocilja

**Affiliations:** 1 Agricultural and Biological Engineering, Institute of Food and Agricultural Sciences, University of Florida, Gainesville, FL 32611, USA or tvlmorgan@ufl.edu (V.M.); ncavallaro@ufl.edu (N.C.); gkiker@ufl.edu (G.K.); yuerong@ufl.edu (Y.R.); 2 MIT Auto-ID Labs, Department of Mechanical Engineering, Massachusetts Institute of Technology, Cambridge, MA 02139, USA; 3 MDPnP Labs, Biomedical Engineering Program, Department of Anesthesiology, Massachusetts General Hospital, Harvard Medical School, 65 Landsdowne Street, Cambridge, MA 02139, USA; 4 Molecular Biosciences and Bioengineering, University of Hawaii Manoa, Honolulu, HI 96822, USA; danielje@hawaii.edu; 5 Mechanical Engineering, Iowa State University, Ames, IA 50011, USA; carmen@iastate.edu; 6 Mechanical Engineering Department, Iowa State University, Ames, IA 50011, USA; jcclauss@iastate.edu; 7 Ames Laboratory, Ames, IA 50011, USA; 8 Environmental Engineering and Earth Sciences, Clemson University, Clemson, SC 29634, USA; dvanega@clemson.edu; 9 Global Alliance for Rapid Diagnostics, Michigan State University, East Lansing, MI 48824, USA; alocilja@msu.edu; 10 Nano-Biosensors Lab, Michigan State University, East Lansing, MI 48824, USA

**Keywords:** sensor, smart systems, data analytics, cyber-physical systems, artificial reasoning tools, ART, drag and drop analytics, DADA, sensor-analytics point solutions, SNAPS, sense-analyze-respond-actuate, SARA, machine-assisted tools, MAT, machine-assisted platform, MAP, knowledge graphs, trans-disciplinary convergence

## Abstract

In this review, we discuss the role of sensor analytics point solutions (SNAPS), a reduced complexity machine-assisted decision support tool. We summarize the approaches used for mobile phone-based chemical/biological sensors, including general hardware and software requirements for signal transduction and acquisition. We introduce SNAPS, part of a platform approach to converge sensor data and analytics. The platform is designed to consist of a portfolio of modular tools which may lend itself to dynamic composability by enabling context-specific selection of relevant units, resulting in case-based working modules. SNAPS is an element of this platform where data analytics, statistical characterization and algorithms may be delivered to the data either via embedded systems in devices, or sourced, in near real-time, from mist, fog or cloud computing resources. Convergence of the physical systems with the cyber components paves the path for SNAPS to progress to higher levels of artificial reasoning tools (ART) and emerge as data-informed decision support, as a service for general societal needs. Proof of concept examples of SNAPS are demonstrated both for quantitative data and qualitative data, each operated using a mobile device (smartphone or tablet) for data acquisition and analytics. We discuss the challenges and opportunities for SNAPS, centered around the value to users/stakeholders and the key performance indicators users may find helpful, for these types of machine-assisted tools.

## 1. Overview

A plethora of literature reviews describe the historical context [1], recent advances [2,3,4], and futuristic ideas [5,6,7] related to development and application of chemosensors, biosensors, physical sensors, and nanosensors [8,9]. These diagnostic tools have important applications across the medical, agricultural, and environmental domains, and in some cases overlap multiple areas. Chemosensors, physical sensors, biosensors and nanosensors (collectively referred to as sensors herein) have enormous potential as point of care (POC) devices [10,11], also known as point of need devices [12,13]. The majority of POC sensor applications are in the medical and public health fields, although recently the library of tools for agricultural and environmental applications has been expanding rapidly [14,15,16]. The expansion of data connectivity within POC devices [17] is a catalyst for divergent application of sensors into otherwise restricted domains. In agricultural and environmental applications, enhancing mobility through data connectivity is paramount, and many current efforts are focused on wireless sensors connected to mobile devices. However, in most cases there are no analytic tools directly embedded into the mobile device and post hoc analysis is required using a laptop or computer. In this review, we discuss select POC systems and introduce sensor-analytics point solutions (SNAPS) as a platform for aggregating sensor data for analytics. SNAPS illustrates a confluence of ideas, including sensing, mobile devices, connectivity and cyber-physical systems, which may be combined with artificial reasoning tools (ART) and data-informed decision support systems. SNAPS serves as a proof of concept for ensuring the appropriate hardware/software tools are matched, ensuring diverse stakeholder needs are matched with sensor data and analytics.

This review provides an overview of sensor engineering related to the recognition-transduction-acquisition triad as it relates to basic design decisions for SNAPS, followed by a discussion of hardware and software elements which may be necessary for SNAPS. We then introduce elements of autonomy, provide specific examples of SNAPS, and we point out a few of the challenges and opportunities. We conclude by discussing the importance of making sense of data and how to deliver information on demand from data to users and stakeholders, before the quality of service perishes, in the context of actionable information which possesses transactional value (see Figure S1).

## 2. Sensor Engineering

Sensor engineering is rooted in material choice, and development of practical protocols that enhance device accuracy without sacrificing temporal resolution. The fundamental sensor working mechanism established by the International Union of Pure and Applied Chemistry in the 1990’s has consistently served as the design backbone for research groups (see Figure S2). The coating on the sensor surface selectively binds the target, a transduction event produces measurable signal, and the signal is acquired using specialty equipment. This sensing process, based on the recognition-transduction-acquisition (RTA) triad, has been enhanced through the use of nanomaterials that improve detection limit, speed and/or reversibility [18,19]. In biosensors, biomaterials are commonly employed to improve selectivity, bandwidth, or facilitate actuation [20,21,22]. Recent progress in engineering nanoscale materials has paved the way for development of non-biological chemical and physical sensors that accomplish some of these same improvements [23,24]. Whether the nature of the recognition event is chemical, biological, or physical, these molecular scale interactions are the initial step in sensing, and the material choice governs the efficacy of this RTA triad.

**Figure 1:**
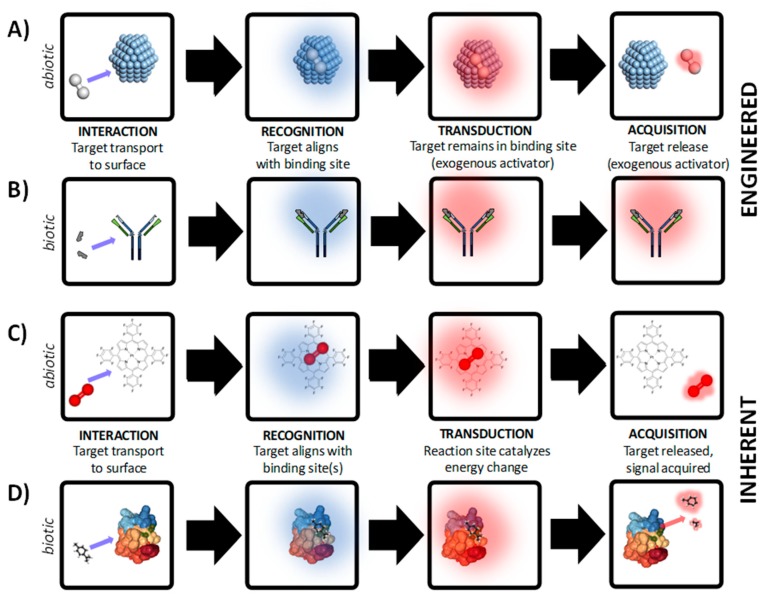
Development of chemical/biological/physical sensors is based on either engineered transduction where an external process is engineered to control transduction and/or acquisition for reversible sensing (panels A–B) or inherent transduction where (panels C-D) where activation energy is supplied by target and ambient environment for reversible sensing. In both examples, abiotic and biotic examples are demonstrated. (**A**) Abiotic sensing of H_2_ (g) with Ag+ particles. (**B**) Biotic sensing in a lateral flow assay based on antigen-antibody interactions. (**C**) Abiotic O_2_ (g) sensing based on the luminescent dye platinum porphyrin dyes. (**D**) Odorant sensing based on chemosensory proteins. Structure of protein in panel D courtesy of Mosbah et al [36]. The examples shown here are for demonstration purposes, and do not represent all chemical, biological, or physical RTA mechanisms.

The affinity of the sensor coating for the target is the limiting factor for device function, and the importance of this first step in the RTA triad cannot be over-emphasized. Given that binding affinity and selectivity are the architects of the RTA triad, transduction is the platform for innovation. Intuitively, material choice dictates classification of device as either a sensor (use of abiotic materials), biosensor (biological or biomimetic materials), nanosensor (nanomaterials), or nanobiosensor (hybrid nano/biomaterials). In addition to establishing these commonplace definitions, sensor material choice dictates critical performance factors such as durability, cost and ultimately quality of service. In its most basic definition, transduction is defined as a change in energy state. There are two major classes of transduction that lead to the evolution of quantitative data or qualitative data, namely inherent transduction and engineered transduction, respectively (Figure 1).

Engineered transduction (Figure 1A,B) involves highly specific binding of the target by the receptor but cannot be used for reversible, continuous measurement due to the need for an exogenous reagent or engineered process for at least one of the following: (i) facilitation of the transduction step, or (ii) promoting release of target from binding site. In either case, the sensor cannot autonomously produce a measurable product without an engineered process or exogenous reagent. In this type of sensor (often referred to as a dosimeter), the thermodynamics do not lead to favorable production of an active compound which can be directly quantified using acquisition equipment. There are generally two situations which require addition of exogenous reagents and/or engineered processes: compound(s) which facilitate a change in activation energy that can be measured, or compound(s) which promote desorption of the target from the receptor binding site for sensor reuse. Examples of exogenous reagent(s) and engineered processes include: fluorescent labels [25], heating elements [26], supporting material(s) in close proximity to the recognition structure such as a coloring enzyme [27], strong acid/base to denature target-receptor bonds [28], among other examples. For example, sensors based on binding between H_2_ (*g*) and Ag^+^ nanoparticles or Ag^+^ films are not reversible without external heating of the sensor surface (Figure 1A). In a biotic example, biosensors based on covalent binding between antibodies and antigens are commonly used in lateral flow assays (Figure 1B). In most cases the binding between the target and receptor material is covalent and cannot be reversed without considerable additional cost. In the case of lateral flow assays, the recognition structure is co-immobilized with a secondary structure that, upon binding of the target, undergoes a specific reaction and leads to a visible color change [29]. In this type of transduction, covalent bonds between the target and recognition structure are typically intact after the signal is acquired, leading to a significant amount of hysteresis. Due to the hysteric binding between target and receptor, devices based on engineered transduction are typically not reusable as attempts to recover the native binding chemistry of the receptor are not known, at this time. There are examples of reversible covalent bonds for sensing based on chiral nematic liquid crystals [30], and other recent work has demonstrated re-usability using allosteric triggers engineered within the recognition mechanism [31,32]. At present, semi-quantitative data can be obtained using engineered transduction approaches, if a sensor array is developed but the hysteric molecular interactions restrict the data from being truly quantitative.

Sensors which autonomously produce quantitative data are classified as inherent transduction (Figure 1C,D). For example, an abiotic sensor based on non-covalent metal coordination between O_2_(*g*) and platinum porphyrin is shown in Figure 1C. An example of a biosensor based on enzyme-ligand interaction is shown in Figure 1D. In this type of transduction, binding of the target by the receptor leads to the production of a measurable by-product with little or no hysteresis. No additional engineering is needed to obtain useful signal correlated to the binding event, as the thermodynamics of the system indicate that the presence of the target alone is the rate limiting step for energy state change. The formation of the product can be directly correlated to the presence of a specific concentration of target, with the product formation well described by stoichiometry. The most common example of inherent transduction is the glucose biosensor for blood analysis, where glucose and oxygen are both present in blood, and serve as activators of the enzyme-catalyzed oxidation due to GOx, or glucose-1-oxidase (beta-D-glucose:oxygen-1-oxidoreductase, EC 1.1.3.4). In this reaction, the oxidation of glucose on the sensor surface results in the production of electrons, which are measured using oxidative amperometry [33]. There are many examples of non-contact sensors which are reusable, such as pulse oximeters for blood O_2_ inference [34] which are critical for vital sign monitoring but lack the specificity of quantitative tools such as the GOx sensor. Optimizing performance tradeoffs between quantitative sensitivity, response time, selectivity, and range is a task which begins with the user in mind [35], and requires a detailed understanding of the problem context prior to design considerations which are based on material choice. One major advantage of inherent transduction over engineered transduction is that the bonds between the target and receptor are inherently destabilized during the transduction process, leading to diffusion of reaction by-products away from the binding site after by-product formation. Reducing sensor hysteresis facilitates development of reusable sensor chemistry, allowing continuous or in line sensing.

Whether the recognition involves a biomaterial, abiotic, or nanophase material, in most cases multiple chemical bonds occur between the target and the receptor material, and the strength of these bonds governs the specificity, limit of detection, response time, and hysteresis of the sensor. Mismatch between material choice and intended application (see Figure S3) results in loss of quality of service, and in some cases a complete lack of technology acceptance. Assays and sophisticated *post hoc* analysis techniques can resolve some of this mismatch, but there are limits. To preserve and elevate the quality of the outcome, selection of appropriate material(s) should be paired with sensing protocols and analytical techniques, discussed in the following section.

### Point of Need Sensing and Smartphones

Point of need sensors are a critical tool for medical, agricultural, and environmental monitoring, and the applications of these tools has been diversifying over the last few decades. The primary application space for point of need sensors has been the analysis of unique targets using relatively low cost, rapid detection platforms [37], including small molecules [38,39], viruses [40,41] and cells [42,43] (amongst other targets). Recent works have focused on enhancing the mobility of point of need sensors for rapid on site applications [44] by limiting the requirement for equipment or *post hoc* methodologies that depend on a formal laboratory. Most portable/handheld sensors are not designed to compete with standard analytical laboratory diagnostics, but rather as a parallel tool to trigger new questions or provide additional sampling to improve resolution. Attempting to use a handheld sensor to produce the accuracy and precision that is commonplace in laboratory-based analytical techniques is in most cases not realistic, and often cost prohibitive. What is realistic, on the other hand, is the development of low cost, light weight, rapid diagnostic tools that can provide point solutions to match the specific context of urgent questions. These urgent questions are posed by millions of people in remote rural communities every day, but from a technology point of view may represent the “lower hanging fruit” from the tree of complex problems. Mobility of customized/personalized sensors in an open-access format may prove to demystify the complexity of certain intractable problems, increasing knowledge while providing service to communities in need, and in turn enabling science to serve society. Mobile phone-based data acquisition systems are primary catalysts for mobility of sensor data in this context [45].

**Figure 2:**
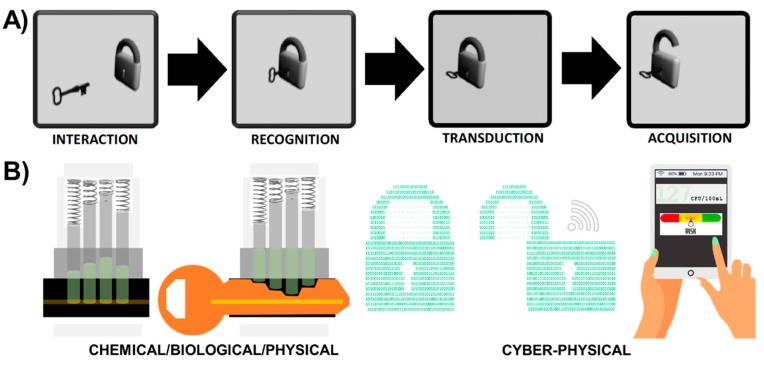
Sensor-Analytics Point Solutions (SNAPS) optimize synergistic integration and connectivity between chemical/biological/physical sensing with cyber-physical systems. (**A**) Classical “lock and key” metaphor for sensor/biosensor/nanosensor design. (**B**) Sensor signal transduction (physical/chemical/biological component) and transmission to a mobile device coupled with in-network processing and on-site edge analytics (cyber component).

Smart phone point of need sensors are available for optical transduction techniques such as fluorescence [46] and surface plasmon resonance [47], in addition to electrochemical transduction techniques such as voltammetry [48] and impedance spectroscopy [49]. While analytical capabilities have grown exponentially in the last decade due to the rapid diffusion of tools such as machine learning [50,51,52], there are only a few examples of mobile phone-based data analysis tools in the literature [53] as most data analysis occurs on computers and not on mobile devices. To maintain the integrity of user/stakeholder needs and ensure quality of service, mobile phone-based sensors may be connected to remote analytics which most modern mobile devices are capable of supporting. SNAPS is a platform approach for transforming sensor data into actionable information using the mobile phone for data acquisition and performing near real-time, on-site, edge analytics on a mobile device such as a smart phone or a tablet (Figure 2).

## 3. Sensor-Analytics Point Solutions (SNAPS)

**Figure 3:**
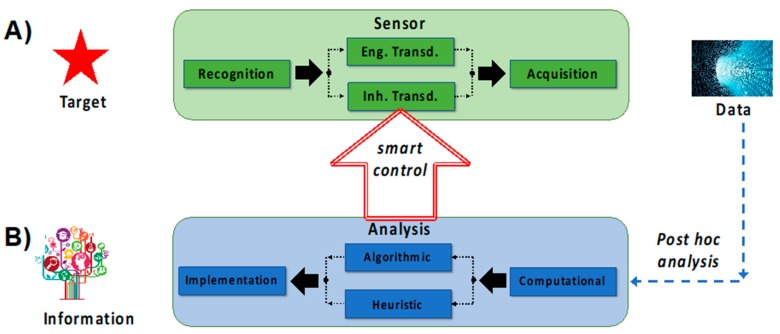
SNAPS attempts to transform data into information based on convergence of two distinct areas, namely sensing and analytics. The framework for these two areas is described by: (**A**) standard sensor development guided by RTA logic, and (**B**) data analysis using the usual tools (computational, algorithmic, statistical). Smart control can be achieved when data from the analysis step actively controls (auto-actuates) at least one process within the RTA sensor triad.

**Figure 4:**
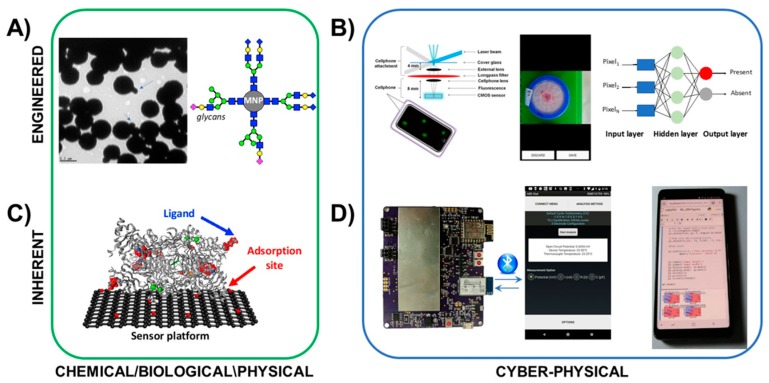
Examples of hardware and materials that may be used for SNAPS for engineered transduction (top) and inherent transduction (bottom). These examples are by no means comprehensive but instead demonstrate the convergence of chemical/biological sensors with cyber-physical systems for delivering computational capabilities and algorithms to sensor data. (**A**) Bacteria-specific magnetic nanoparticles provide dual functions of labeling and enhancing aggregation in detection of target cells. (**B**) Cyber-physical tools for engineered transduction may include smartphone-based microscopes for cell imaging, with the images processed by embedded algorithms for informing decision support. (**C**) Oxidase-based biosensors enable inherent transduction, limiting hysteresis and enabling reusability. (**D**) Handheld potentiostats may be used to acquire electrochemical sensor signals, and embedded or cloud-based analytics such as SVML tools (Reproduced from [53] with permission from The Royal Society of Chemistry.). may be used to provide analysis and decision support. Photograph of glycan-functionalized magnetic nanoparticles courtesy of Bhusal et al [59]. Crystal structure of diamine oxidase courtesy of McGrath et al [68]. Fluorescent smartphone sensor platform courtesy of Wei et al [61]. Image of colorimetric *E.* coli test and machine learning knowledge graph from Gunda et al [66].

SNAPS consist of a biological/chemical/physical sensor directly interfaced with an analytical tool on a mobile device. The general concept of sensors using mobile devices is not new (for example see review by Quesada- González and Merkoci [45], but to date this may be the first review that focuses on convergence of sensing and analytics on mobile device platforms with an equal balance on the two domains. In this section we provide a roadmap for matching transduction type to analytics (Figure 3) and then we review a select number of recent advances in hardware and software used for SNAPS (Figure 4). The green box in Figure 3A summarizes the RTA triad and displays a choice between the two types of transduction discussed in Section 2. Once a receptor material is selected and the appropriate transduction scheme is engineered, the process is coupled with acquisition equipment to obtain signal (data). The blue box in Figure 3B shows the post hoc data analysis phase of SNAPS, which aims to extract actionable information from sensor data. Contrary to the standard used in sensor design, the analysis phase is less standardized, primarily due to lack of platform(s) for data diagnostics, data quality, context, problem space, and query semantics [54]. As an example of a common framework, Marr’s framework is shown, which is a learning principle grounded in Bayesian inference. Marr’s analysis process flow has three interconnected steps: (i) a computational stage, (ii) an algorithmic or heuristic step, and (iii) an implementation step [55]. Analogous to the two types of transduction previously discussed in Section 2, the choice of a heuristic or algorithmic approach should be directly linked to the problem context in order to maintain quality of service (QoS). The implementation step deconvolutes processed data using a relevance filter for producing actionable information. An important *a priori* consideration for SNAPS is that the context of the problem should drive the design of both the sensor performance and the type of analytics to extract information.

In advanced SNAPS, the analysis phase may have an optional feedback control loop with the sensor transduction step, which may be referred to as smart SNAPS. For example, the temperature, pH, electrical potential, or light intensity can be modulated to influence the sensor transduction step based on information obtained from the data analysis phase. Active control of any phase in the RTA triad qualifies as a smart SNAPS but interfacing with the transduction step is the most logical route for adding value. This concept is broadly referred to as sense-analyze-respond-actuate (SARA), which offers tremendous opportunity for controls systems (a detailed discussion is beyond the scope of this manuscript). In SNAPS, acquisition and analytical processing occurs at the edge by deploying a mobile platform of tools using a smartphone or a tablet, or other similar devices as mobile hosts. The next section demonstrates a few examples of these hardware and software tools in the current literature.

### SNAPS Hardware and Software

Figure 4 shows an example of the hardware and materials that may be used for the development of SNAPS. There are many other examples in the literature [46,47,48,49], but these two cases overview engineered and inherent transduction as proof of principle. There are a myriad of other approaches for optical smartphone sensing [47,56,57] as well as electrochemical sensing [48,58], and each has value. The examples in this review are by no means comprehensive (see Table S1 for summary).

An example of engineered transduction (top of Figure 4) demonstrates engineered transduction for diagnosis of tuberculosis (TB) via detection of acid-fast bacilli in sputum samples. Biorecognition is grounded in principles of glycobiology, where target cells are labeled by glycan-coated magnetic nanoparticles (GMNP) [59,60]. In this example, a neodymium magnet is used to separate the particle-cell aggregates to facilitate rapid determination of acid-fastness and cording properties of captured mycobacteria. The TB test also employs Gram staining (an irreversible process) to provide visual confirmation. Smartphone-based optical systems such as the device by Wei et al [61] or the complex microfluidic system by Zheng et al [62] may be used for expanding on-site image analysis, and image processing algorithms [63,64,65,66], may be used for improving accuracy and providing decision support, among many other similar image acquisition algorithms.

An example of inherent transduction (bottom of Figure 4) is demonstrated for detection of biogenic amines using a the graphene-diamine oxidase nanobiosensor developed by Vanegas et al [67]. In this example, an enzymatic biosensor was developed based on diamine oxidase, which was tethered to a laser scribed graphene electrode (LSG) decorated with nanocopper. Upon recognition of the target ligand within the enzyme binding pocket, oxidation is carried out to produce hydrogen peroxide as a by-product. The peroxide is then deprotonated under an operating potential of + 500 mV to produce electrons, measured using oxidative amperometry. Signal acquisition is conducted using a handheld potentiostat connected to a mobile phone such as the ABE-STAT tool developed by Jenkins et al [49]. Further, the support vector machine learning (SVML) classification system developed by Rong et al [53] may be applied using the same mobile phone via the Jupyter notebook open source machine learning tools.

Sensor data, and thus the hardware to collect the data, are core competencies required to fuel SNAPS or any equivalent tool in the portfolio of machine-assisted tools (MAT). Without data, subsequent progress from SNAPS to decision support tools is impossible. SNAPS require the point of need (i.e., mobile) platform to have near real time access to data analytics, statistical characterization and algorithms via embedded systems in mobile devices, or sourced, in near real-time, from mist, fog or cloud computing resources. In the next section we review recent advances in software applicable for SNAPS.

**Figure 5:**
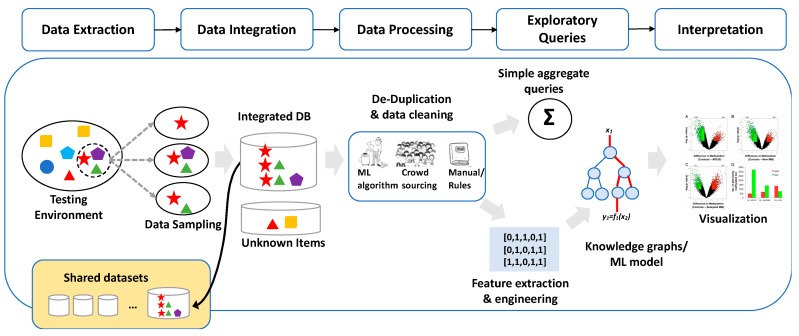
Example of software tools such as drag and drop analytics enable cloud-based analytics for SNAPS. The tool shown here automatically quantifies the different types of uncertainty/errors within data exploration pipelines (image from Chung et al [78] modified to match context of SNAPS). Diagram shows workflow (**top**) and an example pipeline (**bottom**). DB = database, ML = machine learning.

A wide range of commercial and custom software are available for cloud-based analytics [69,70,71], and the list of tools is growing. SNAPS may deliver actionable information through contextually relevant applications using combinations of machine-assisted tools (MAT) and machine-assisted platforms (MAP), enabled by user friendly innovative tools such as drag and drop systems. Drag and drop analytics [72,73,74,75,76,77] may be quite useful in this context. One example of drag and drop analytics is the tool developed for quantifying uncertainty in data exploration (QUDE). QUDE automatically quantifies different types of uncertainty/errors within data exploration pipelines [78]. The automation feature in this tool is based on the following workflow: data extraction, data integration, data processing, exploratory queries, machine learning, and finally interpretation. QUDE is not intended to represent a global solution for all problems relate to SNAPS, but rather demonstrates one approach that may serve as a starting point to connect sensors to analytics in real time based on the intuitive drag and drop interface. While this basic concept is clear, evolution of data analysis from extraction to visualization, even in a drag and drop *modus operandi*, is a process which requires a deep understanding of the context and is highly problem specific. Visualization tools (such as the volcano plots in the right side of Figure 5) are information-rich presentations of complex datasets which may facilitate use of a tool in multiple application domains, but the information is typically not comprehensible to users/stakeholders. Knowledge graph algorithms, in combination with statistical analysis and machine learning (for example, feature engineering, extraction and selection) [79,80], are elements likely to improve this aspect and facilitate evolution of the tool to enable data-informed decision support. This gradual evolution of SNAPS towards a higher order tool supports advanced features such as data-informed decision as a service (DIDA’S), as discussed in Section 4 and Section 5.

The plethora of tools reviewed here and elsewhere may operate in harmony in specialized facilities (such as academic research centers). However, the real value of the convergence is at the hands of the end-user, who may lack specialized knowledge of software or systems. Without lucidity as a guiding light in the design process, the applications of these tools may never be realized outside of research projects. Adoption largely depends on creating user interfaces no more complicated than a menu of choices, for example, the type of variant configuration (i.e., dashboard) that enables a user to customize a laptop. Thus, the paradigm of “plug and play” must be at the front and center of this discussion in order to hide the complexity behind simple drag and drop features which will empower the end users to efficiently interact with the tools [81,82]. Simplified drag and drop tools for SNAPS will exponentially accelerate the global demand for these sensor tools. Democratization of access through Lego-esque modular drag and drop interfaces [83], may pave the way for mobile decision support systems and partial autonomy. Drag and drop analytics coupled with SNAPS has enormous application potential, not only in the agro-ecosystem but in any domain, for example, healthcare, manufacturing [84], finance, utilities, logistics, transportation and retail. In the next section we discuss opportunities for partial automation of SNAPS.

## 4. Auto-Actuation and Partial Levels of Autonomy for Low-Risk Automation

In this section we introduce concepts of autonomy as they relate to SNAPS and discuss future possibilities for partial levels of autonomy in SNAPS. Autonomy is a framework that emerged from intelligent control and systems theory which dates back at least a half century [85]. The specific sensor need and problem context, predicate the architecture of the autonomous system (including both hardware and software). Not all sensors or sensor systems are required to be involved in higher levels of autonomy and there are many problems which only require partial autonomy [86,87]. For example, the purpose, architectural details, system functions, and characteristics for unmanned terrestrial vehicles are different compared to unmanned space vehicles [88]. Although these differences amongst different sensor systems are clear, one unifying attribute is the need to detect a target (sensors on the front end of the process) and then analyze the data (analytics on the back end) in near real time. Lessons from automation in the automobile or aerospace industry, among others, may serve as a knowledge base for engineering and optimization of SNAPS to deliver value, albeit in a very different context and with different specifications.

In the context of SNAPS, the traditional six levels of autonomy (see Figure S4), may inform design and serve as a guide. In the lowest level of autonomy (simple), human interaction is required for direct control of the sensor system(s) and/or manual off-loading of data for post hoc analysis. For example, deployed buoy systems are common in environmental studies of aquatic chemistry [89], which represents the current state for most sensor data. In the second level of autonomy (assisted), a high degree of human interaction is required, but at least one aspect of SNAPS (either sensing or analytics) is capable of performing task(s) without *de novo* synthesis of a pathway map. These tasks may achieve prescribed objective(s), adapt to environmental changes, or develop new objectives. For example, Rong et al [53] recently developed an open source mobile-phone based analytics protocol for analyzing impedance data acquired from a nanobiosensor without *a priori* knowledge of sensor type. The primary objective of the tool is to perform the first layer of analysis in development of a SVML classifier to analyze impedance data (in lieu of equivalent circuit analysis). The mobile phone-based tool automates selection of classifier type using principal components analysis, and subsequently automates selection of hyperplane parameters (optimizes the support vector classifier and support vector regressor functions across the selected hyperplane). While this MAT does not perform decision support or provide validation layer(s), it may qualify as machine-assisted automation, particularly if the impedance data is acquired using the same hardware and the sensing/analysis processes are linked for on-site edge analytics.

The classical third level of automation, partial autonomy, may be achieved through remote control of SNAPS related features, including sensing, data download, and some form of data analytics such as heuristic risk assessment. The outcome may trigger a low-risk set of logic tools to execute a workflow which sets into motion an auto-actuation function. By embracing and accomplishing auto-actuation, the concept of SNAPS marches forward to merge with the principle of SARA for enabling auto-actuation. For example, SNAPS may auto-adjust the water flow rate in irrigation pumps (by temporarily overriding a pre-set routine flow rate) based on updated moisture data from field sensor(s) and refreshed external weather data. Hence, smart control systems like SNAPS and SARA are derived from principles of bio-mimicry because feedback control (activation/inhibition) is the bed-rock of biological systems in maintaining homeostasis and cellular equilibrium [90].

Higher levels of automation may exceed the scope of SNAPS. If the desired outcome of a sensor involves some element of auto-actuation or partial automation, the principle of auto-actuation suggests that we must integrate elementary logic layers, relevant to the context of the event, to enable SNAPS to execute the action using/combining a set of contextually relevant output from SNAPS. For partial automation, SNAPS shall increasingly rely on integration of logic structures, for example, integrating output from SNAPS in decision support for auto-actuation. Integration of logic in the SNAPS architecture indicates a departure from simple point solutions and an upstream move toward higher levels of autonomy, a layer of convergence beyond this review.

In the next section, we focus on two major categories of SNAPS: (i) sensors with engineered transduction coupled with heuristic analysis of qualitative data, and (ii) sensors with inherent transduction coupled with algorithmic analysis of quantitative data. This organization into two categories is designed to meet user needs while maintaining an appreciable quality of service. 

## 5. Coupling Sensor Transduction with Data Analytics for Decision Support

In this section we present two generic cases of SNAPS, each case is intended to match sensor transduction type with the appropriate class of analytics based on the logic in Section 2 and Section 3. In the first case, qualitative or semi-quantitative sensors (engineered transduction) are matched with qualitative (i.e., heuristic) analytics. In the second case, quantitative sensors (inherent transduction) are matched with quantitative (i.e., algorithmic) analytics. While these two cases are not intended to cover all possibilities, we discuss the importance of ensuring that sensor data and analytic tools are appropriately coupled. In Section 6 we provide a tangible example of each type of SNAPS.

**Figure 6:**
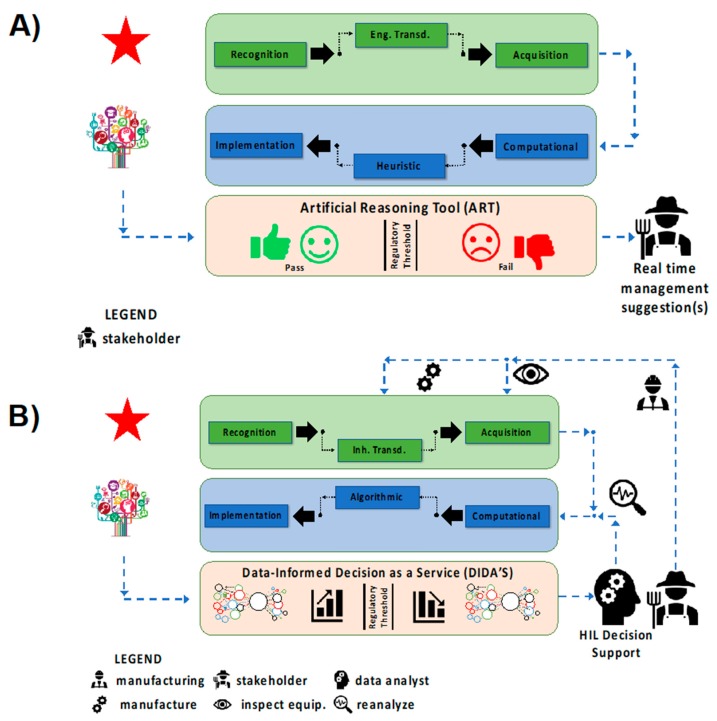
Classification of two major types of SNAPS based on user/stakeholder expectations. (**A**) SNAPS-ART produces qualitative, or semi-quantitative sensor data based on engineered transduction for analysis using heuristic analysis tools to provide management suggestions. (**B**) SNAPS-DIDA’S produces quantitative, streaming data for algorithmic analysis to be implemented in a variety of decision support paradigms, which may include human-in-the loop and/or agent-based systems. The diagram provides a basic map for avoiding mismatch (i.e., loss of quality of service) between sensor chemistry and application needs but are not intended to be dogmatic.

The first category of tool (Figure 6A) utilizes qualitative sensors based on engineered transduction coupled together with heuristic analysis to produce artificial reasoning tools (ART). This category, deemed SNAPS-ART, is designed to provide near real time management suggestions, such as the use of a single sensor to determine whether a particular sample is above or below a threshold set by a regulatory agency. The assumption of high fault tolerance and low risk are pivotal to development/deployment of SNAPS-ART. To maintain quality of service while optimizing development costs, acquisition of qualitative data for SNAPS-ART uses engineered transduction techniques and heuristic classification to satisfy user expectations with a binary output (for a rapid YES/NO test). In terms of active control features, the SNAPS-ART platform may be quite rudimentary, with only a few discrete and distinct actions (turn on/off a subsystem) determined by simple non-overlapping binary outputs based on input from SNAPS. SNAPS-ART is not intended to be a comprehensive diagnostic tool, but rather designed for triage or rapid screening, where additional testing is often required to confirm/validate results. It is possible to use ART for semi-quantitative purposes that depends on other combinatorial factors (e.g., flowrate control), but within reason. The layer of ART may be conceptually viewed as a holding platform for machine-assisted tools, which apply basic pseudocode with simple logic to provide an output sufficient to execute a low risk action which is highly fault tolerant.

The second category of tool (Figure 6B) utilizes quantitative sensors based on inherent transduction coupled with algorithmic analysis for providing data-informed decision as a service (DIDA’S). The tools are collectively referred to as SNAPS-DIDA’S. Contrary to SNAPS-ART, this category is designed for decision support under the assumption of low fault tolerance and moderate risk where real time, continuous monitoring is required. Rather than instantaneous results that are classified by heuristic data analysis techniques, a defining feature of SNAPS-DIDA’S is the dynamic/reiterative analysis of streaming data from sensors as well as feedback logic that interfaces with processed data. For example, active control features using a case-specific subset of tools from a super-set of MATs and MAPS (machine-assisted tools and machine-assisted platforms, respectively). Optimization based on menus of choices and ranges of values, for each variable, require computational rigor to extract context-specific variant configurations rather than workflow middleware as the control layer. Sophisticated decision support software with decision trees executing embedded logic is one option that may be user-directed [91,92]. The latter may be enabled by a drag and drop assembly from the portfolio of modular tools under the umbrella of MAT and MAP. Another option is to present these choices to a human-in-the-loop who may exercise some form of exclusion/reduction to narrow the search space (number of choices) from the MAT/MAP menu based on experience and knowledge [93,94]. The third and the preferred long-term option is the development of a parallel agent-based system (ABS) which may be part of a multi-agent system (MAS) [95,96]. The agent is expected to be highly specific for certain pre-determined functions and endowed with the capability to replicate (reason) a few of the elementary choices and selection functions as if resembling the human-in-the-loop. ABS cannot benefit directly from human experience and/or human ability to handle exception management, which restricts the range of options to the arsenal of information and logic rules that are embedded into the ABS. One of the major limiting factors is the inability to train a software agent and invoke actual learning, especially regarding decisions such as how and when to use a particular tool. Due to the cognitive boundaries of deterministic design, it is not possible to for a training tool or machine learning routine to educate an agent to deliver support in non-deterministic scenarios. The latter makes it mandatory to recognize the boundaries of “artificial” systems and consider maintaining provisions for humans-in-the-loop, by design, for non-deterministic cases (exception management).

The theoretical boundary between ART and DIDA’S is blurry, at best. The classification of the two systems into discrete boxes in Figure 6 is by no means intended to be reductionist, rather this strategy is merely an attempt to introduce SNAPS and suggest future improvements and innovations. The distinction between ART and DIDA’S may be made in terms of the data that must converge or the degree to which data fusion may be necessary when rendering the decision or recommendation. ART is expected to be a rapid-response system which aims to solve low risk problems with only a few data sources and data dependencies using either qualitative or quantitative sensors matched with heuristic analysis. To contrast the two, qualitative SNAPS-ART may provide the instruction to turn off the irrigation water pump if [a] the rate of change of 80% of the soil moisture sensor readings fall above/below a given range of values or [b] if the data from the sensor(s) indicates that the rainfall rate is above a certain value. For quantitative SNAPS-ART, the instruction may be to monitor and turn up/down the rate of irrigation water flow, by grids/zones, depending on the soil moisture sensor readings, if the sensor data falls above/below a range of values. The tool may refer to the logic instructions in a look-up table which recommends water flow rates versus soil moisture. DIDA’S may be viewed as a mutiny of multiple ART units, each vying to contribute data. At each gateway or node in the DIDA’S platform, there are agents which are queueing, to be triggered by a specific data strand/stream, to initiate a search and discovery process for identifying what tools must be used. This represents dynamic composability of tools triggered by data in a manner similar to application-dependent-networking, which connects two mobile phone users in diverse environments. In addition, agents are triggered to discover which databases or data resources must be accessed, to satisfy the context of dependencies, and when/how to feed the results from the search and discovery to a higher-level agent.

In the context of SNAPS-DIDAS, agent-based systems begin to function upon receiving input from SNAPS. Using logic capabilities (learned, trained, reinforced), agent(s) determine which tool, or sets of tools, may be necessary to execute the action or automation that the SNAPS output expects to trigger. Agents are limited by the tools contained within MAT and MAP, unless embedded logic provides the option to place a remote function call (RESTful API) to a cloud repository to source other tools or algorithms. If this feature is included, agent(s) can “discover” which are contextually relevant for the use case, providing higher levels of automation. Search and knowledge discovery functions of machine-assisted systems are key performance indicators (KPI) which are inextricably linked with quality of service (QoS). Synergistic integration with external tools and modules is subject to interoperability among platforms, which are influenced by standards and architecture. As is apparent from this brief discussion, automation of SNAPS-DIDA’S is far from trivial, and we are only beginning to scratch the surface in terms of the confluence of ideas necessary to transform this vision into reality. In the following section we provide tangible examples of SNAPS, with a specific focus on SNAPS-ART (tangible examples of SNAPS-DIDA’S is beyond the scope of this review).

## 6. Proof of Concept SNAPS

In this section we show two proof of concept SNAPS-ART tools to demonstrate the application of the concept to environmental and agricultural problems related to safe drinking water and food. These two examples were selected based on the global significance of the problem, as well as the transdisciplinary nature of the question at hand for ensuring planetary health (i.e., the health of the planet and the humans that inhabit the space) [97,98,99]. In each case the analytics are embedded into the mobile device and the tool supports manual data entry as well as auto-upload of sensor data.

**Figure 7:**
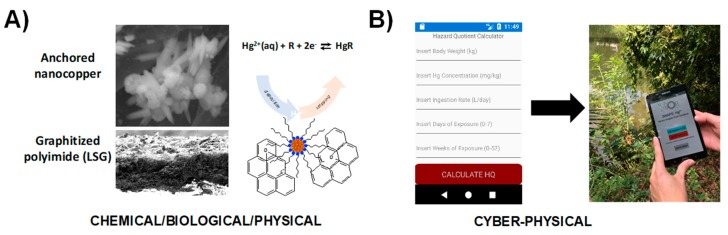
Proof of concept demonstration for SNAPS-ART in heavy metal analysis (drinking water). (**A**) Hg^2+^-selective nanosensor based on LSG and nanocopper developed by Abdelbasir et al [101]. (**B**) Screenshot and photograph of heuristic analysis tool for calculating risk of mercury exposure (hazard quotient calculator).

Figure 7 demonstrates an example of SNAPS-ART for heavy metal analysis coupled with hazard analysis risk assessment. In this design, a sensor with engineered transduction (qualitative data) is coupled with a heuristic risk analysis tool (hazard quotient indicator) for monitoring and assessing risk of mercury exposure in drinking water applied to locations lacking adequate water management infrastructure. The tool was designed for use in rural settlements located near artisanal and small-scale gold mines [100]. In this example, a nanosensor was developed based on LSG electrodes decorated with anchored nanocopper for measuring ionic mercury (Hg^2+^) via stripping voltammetry [101] (Figure 7A). Rapid screening of water samples for mercury contamination is highly useful, but the value of sensor data is inconsequential without information on how compounded factors, such as body weight, ingestion rate, and length of exposure contribute to overall public health risk for an individual. A mobile app was developed in R language (see supplemental section for code) using the heuristic hazard quotient (HQ) methodology used by regulatory agencies across the globe [102]. MIT App Inventor was used to create the smartphone app, which is rooted in drag and drop techniques using the Blockly modular tool for functionality. Figure 7B displays the graphic user interface and an example output for the SNAPS-ART tool, where users input drinking water ingestion rate, body weight, length of exposure, and age. The app captures Hg^2+^ levels (ppm) obtained from the sensor, and uses the framework established by the US Environmental Protection Agency (EPA) and the World Bank to calculate a HQ score [103,104,105]. Using the standard HQ threshold set by the EPA [106], HQ scores greater than 1.0 indicates higher risk of potential adverse health effects increases, while a score less than 1.0 indicates low risk. SNAPS-ART provides a real time management suggestion to the user based on international standards for mercury contamination of drinking water. In addition, ART provides a suggestion to seek additional screening at a verified laboratory if the sample is positive, which is a critical feature for secondary validation. The HQ output significantly increases the end-user value of the sensor by combining the raw sensor data in the context of human-specific factors and micro-environment, to provide actionable information relevant to precision public health.

**Figure 8:**
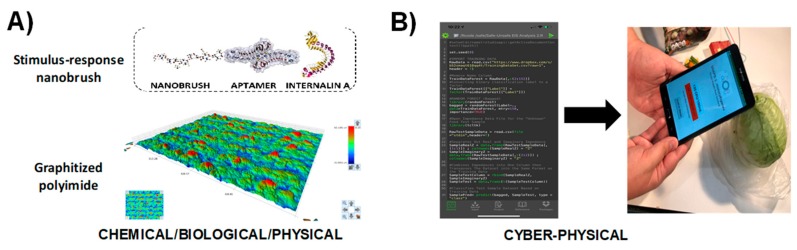
Proof of concept demonstration for SNAPS-ART in food safety analysis (vegetable broth). (**A**) *Listeria monocytogenes* biosensor developed with stimulus-response polymers and DNA aptamers by Hills et al [69]. (**B**) Screenshot and photograph of machine learning analysis tool for determining whether sample is contaminated based on an index score derived from machine learning analysis.

Figure 8 shows another application of the SNAPS-ART platform applied to impedimetric sensors for detection of pathogenic bacteria in food samples. The *Listeria monocytogenes* biosensor developed by Hills et al [107] is used as a demonstration (Figure 8A), and an ART tool was developed using machine learning (code written in R programming language, see supplemental section for code). The ART tool for *L. monocytogenes* detection is grounded in binary classification using bagged random forest, and the smartphone app was created using MIT App Inventor. The program reads a raw impedance data file from the biosensor, converts the data to the necessary form for machine learning classification, optimizes hyperparameter values, and then uses machine learning techniques to compare the sample to a training library; other methods are feasible as described by Rong et al [53]. The tool is used for predicting whether the food sample may be contaminated or is safe according to thresholds set by guidelines established in the Food Safety Modernization Act (FSMA), and how the user may seek secondary validation if the sample is positive (Figure 8B). The major benefit of this tool is the avoidance of computationally expensive (and time intensive) analytical methods such as equivalent circuit analysis. While some equivalent circuit models, such as the Randles-Ershler circuit, provide some description of the physical meaning for each circuit element related to an impedimetric biosensor, in most cases more complex models are used and parameters are tuned with Chi^2^ fitting. If not used with expert guidance, equivalent circuit analysis leads to significant errors in both interpretation and accuracy and may be cost prohibitive for many labs. In field analyses, equivalent circuit analysis is computationally and energy intensive, limiting the practicality for monitoring rural regions or dense urban areas where network connectivity and power are limited. For pathogens such as *L. monocytogenes*, the threshold for contamination is one live cell in a food sample, and thus speed and accuracy of the tool are paramount, particularly for rapid screening. Delays in data analysis lead to food waste, increased risk of contamination, and a significant reduction in quality of service [108]. Use of machine learning tools and other similar algorithms to rapidly perform screening on site with SNAPS significantly increases the value of the sensor and provides actionable information in the hands of the user, regardless of location or access to a formal laboratory.

The benefits of the approach in Figure 7 and Figure 8 may be vastly extended by adapting the sensor RTA scheme to allow the tool to detect and alert users about other targets. For example, development of a sensor array to simultaneously target other heavy metal contaminants including lead, cadmium, and arsenic or other biomolecule targets such as viruses. SNAPS-ART may be used for detection and diagnostics for a plethora of contaminating agents not only in liquid or food (as shown here), but in any other medium as long as the analyte is presented in a form that binds with the sensor material. The immense value of this approach when combined with mobility (smartphones) is the ability to source sensor data for a myriad of analytes in different environments where humans or drones may reach to interact with the sample. In our approach, we have eschewed the use of high cost sensors, to highlight the potential for diffusion of low-cost tools to enable democratization of data and distributed decisions to serve community-specific needs. To acquire, curate, analyze and extract useful information from sensor and other data, we advocate synergistic integration with MAT and MAP. SNAPS is a preliminary step in that direction, and there are a many challenges and opportunities as discussed in the following section.

## 7. Challenges and Opportunities

### From SNAPS to PEAS

There are many challenges and opportunities for SNAPS (see Table 1). As SNAPS evolves, sensor engineering for controlling or modulating hysteresis is an absolute requirement if the user expectation is rooted in real time, in situ, sensor data connected to data analytics (see Figure S5). Rudimentary control over system performance through the use of SARA-driven smart SNAPS to auto-actuate select system components is applicable to the agro-ecosystem, environmental health, as well as public health. While no specific example is shown here, SNAPS is the first step toward DIDA’S, which may rely on tools such as drag and drop analytics and models using agent based systems. DIDA’S is a tangible goal on the horizon, but current progress is rather slow. In agricultural and environmental systems, connectivity is often assumed, but rarely functional at the level required for a complex system [109,110,111] such as SNAPS-DIDA’S. As SNAPS and similar tools mature, the true value may be realized through the interaction of agents which embrace the collective optimization of performance in the context of the environment. This futuristic concept is captured by the convergence of performance metrics (precepts, environment, actuators, sensors) or PEAS, a mnemonic borrowed from the literature on agent-based systems (ABS) to address “whole” system performance [112].

There is an enormous opportunity to develop decision support systems using the PEAS as a platform. PEAS are pillars on which we may build “machines that work for us” versus “cogs” in the wheel as envisioned by Ellul a half century ago [113]. SNAPS, ART, and DIDA’S are examples of tools with which we are working and represent short-term opportunities for Pareto-like solutions. Beyond SNAPS, PEAS represent goal-dependent strategic perspectives for systems-level synergy. Each platform contains an array of dynamic push-pull elements and user-directed levers, which may be used in any combination, to accomplish short term tasks (SNAPS) for establishing the foundation of long-term attempts to orchestrate systems performance (PEAS). This interrelationship may be analogous to components of the engine (SNAPS, ART, DIDA’S) which are essential and dependent for the function and performance of the “whole” vehicle (PEAS).

Aggregating data and information for systems performance using the PEAS concept is the Holy Grail and, in some instances, the “whole” picture is the only relevant picture. This concept may seem far reaching, but related attempts in biomedical engineering have already proven viable, such as the integrated clinical environment (ICE) effort [114]. ICE drives data interoperability between all sub-systems to focus on the “whole patient” rather than isolated parts. These two concepts (PEAS, ICE) may serve as a guiding light for innovations applicable to agriculture, environment, or other verticals areas, where a tapestry of solutions may be more valuable than point solutions. Regardless of the application domain, convergence of solutions to create systems level performance is the key challenge going forward (i.e., avoidance of isolated solutions). Isolated solutions in the medical systems lead to errors in medical device interoperability. The latter is often fatal, claiming as many as 250,000 lives per year, in the US alone, and is the third leading cause of death in the US [115]. In the coming decade(s), there is a major opportunity to develop platforms such as PEAS based on the lessons learned from ICE. Integrating such platforms will both improve knowledge gain, as well as contribute to transformational convergent thinking such as the planetary health concept [98,116]. One of the underlying themes in all use cases is the use of mobility and low latency signal transmission (for example, future potential for use of 5G) as key enablers for facilitating various levels of partial autonomy within system of systems, which responds to remote instructions and other relevant secure signals. However, often, very small amounts of data and/or information, at the right time, can be far more critical and helpful, rather than a deluge of data (erroneously referred to as big data).

The excruciating struggle to extract information from sensor data (if there is information in the data) is an indication that unleashing knowledge from information is an enormous challenge, at present. The much-anticipated evolution of data-science to knowledge-science is the central thrust of knowledge-informed decision as a service (KIDS), an aspirational idea which may not be addressed by current tools and contemporary thinking. The broad spectrum of “data-informed” approaches will vary by use cases, from simpler instances where SNAPS-ART may be the first step, to more complex expectations where DIDA’S will be necessary as a foundation. The first step in resolving this immense challenge is to identify which tool is needed, when it is pertinent, and where to apply the tool.

## 8. Concluding Remarks

The pivotal role of sensors, data, and information in decision support and partial automation or auto-actuation is of critical importance in any field. SNAPS represent a confluence of ideas and is the foundation for making sense of data and adding value to sensor data. Basic sensor design choices, described in this review, dictate the value and quality of service for SNAPS, and this fundamental concept cannot be overlooked without inducing a fatal flaw that limits the usefulness of downstream cyber-physical systems. At the most basic level, matching the type of sensor transduction (engineered or inherent) together with the appropriate analytical approach (heuristic or algorithmic) to meet the needs of the end user ensures a baseline quality of service. SNAPS and ART offer a glimpse of a few elements of the machine-assisted tools and machine-assisted programs in terms of the quest to deliver value from data analytics. Drawing on these trends, we suggest how SNAPS may evolve to inform knowledge gain as the system complexity increases.

Knowledge discovery cannot be treated as a separate topic when discussing sensors and sensor data. Without discovering the context and relevance of data to the bigger picture, the outcome will remain narrow. Sensors will be impotent without tools to extract value from sensor data. This discussion, therefore, is not peripheral to sensors, it is central to all sensors. The growing ubiquity of sensors which are increasingly woven into almost every facet of our daily lives makes it imperative that sensor scientists and sensor engineers consider the data science impact of their work. Data scientists must take a closer look at sensor data and sensor engineering, to ask the correct questions. Each group must ensure that the tools are designed with the end user in mind for ensuring quality of service. Tools for knowledge discovery are not in short supply, but the rate limiting factor preventing the diffusion of these tools are rooted in their complexity, lack of standards and common open platforms that users can easily access. When and if these “open platforms” emerge, the race to adapt and adopt will not be determined by its success due to technological strength or computational excellence. Rather the economics of technology may be the single most important criteria which will influence and determine feasibility of mass adoption, the latter, in turn, will reduce cost of adoption due to economies of scale.

We are on the brink of change, albeit slowly, with the advances in search and discovery of data and information, using tools based on graph theoretic approach, to establish relationships and dependencies between data, objects, and subjects. New research at the nexus of natural language processing, linguistics, and semantics may be the trans-disciplinary convergence necessary to advance knowledge discovery from sensor data. Broad spectrum dissemination of this knowledge using simple and tangible user interfaces (TUI) will be crucial. Knowledge discovery is at the heart of sensor research and sensor engineering, if we wish to extract value from sensors and aspire to deploy sensors as global public goods.

## Figures and Tables

**Table 1:** Challenges and Opportunities for SNAPS.

**Challenge**	**Opportunities**
Extraction of information from sensor data for real time decision support	Development of SNAPS-ART tools using established regulatory standards as a guide
Controlling or modulating sensor hysteresis in situ	Integration of smart materials on sensor surface (e.g., stimulus-response polymers) Rudimentary control over system performance through the use of sense-analyze-respond-actuate (SARA) systems
Mobility and connectivity in agricultural and environmental systems	Deploy high bandwith, low latency, systems Develop low power sensor data management
Integrating SNAPS into a standardized platform	Establishment of data management systems based on lessons learned from other systems such as integrated clinical environment (ICE) Establishment of standard architectures for real time sensing (interoperable with other standards)
Development of data informed decision as a service (DIDA’S)	Establishment of SNAPS-ART as a common tool Integration of drag and drop analytics (DADA) and agent based systems (ABS) Dynamic/reiterative analysis of streaming data from sensors (captured in time series databases) Demonstration of feedback logic that interfaces with processed data Dynamic composability of tools triggered by data (application-dependent-networking) Database discovery or data resource discovery by agents using embedded logic (e.g., remote function call, RESTful APIs) Use of logic capabilities (learned, trained, reinforced) and agent(s) to determine which tool, or sets of tools, are required for the given problem

## Supplementary Materials

The following are available online at https://www.mdpi.com/1424-8220/19/22/4935/s1, Figure S1: Overview of Review, Figure S2: Recognition-Transduction-Acquisition (RTA) triad, Figure S3: Design of SNAPS must use a retrosynthetic approach, beginning with the intended application in mind. This allows proper selection of materials, transduction techniques, and analytics for ensuring quality of service. (A) Correct matching of engineered transduction with heuristic analytics. (B) Incorrect matching of engineered transduction with algorithmic analytics leads to overdesign of the tool, consuming unnecessary energy and computational power. (C) Incorrect matching of inherent transduction with heuristic analytics leads to excessive data collection, which causes systematic negative effects unless the data. This approach is valid for long term monitoring programs, but is not relevant for rapid, point of need SNAPS. (D) Correct matching of engineered transduction with algorithmic analytics, Figure S4: Traditional autonomy may not be the dogma for development of SNAPS, Figure S5: (A) Information hierarchy depicting the evolution of sensor data towards knowledge. Higher levels (wisdom, understanding) may be beyond the scope of sensor data, but here we describe a platform for evolution of sensor data to information (SNAPS-ART and SNAPS-DIDA’S) and suggest a path forward for evolution to knowledge via the KIDS platform. (B) When KIDS is applied to the agro-ecosystem, the convergence of performance metrics, environment, actuators, and sensors (PEAS) encompass the platform through agent-based systems, Table S1: Recognition-Transduction-Acquisition (RTA) triad.
