# Supplementary material for: SNAPS: Sensor Analytics Point Solutions for Detection and Decision Support Systems
Source: Sensors (Basel). 2019 Nov 13;19(22):4935. doi: 10.3390/s19224935 (PMC6891700; doi:10.3390/s19224935)
Supplement: Supplementary file 1 [file sensors-19-04935-s001.pdf]

sensors

MDPI

Supplemental

Table S1. Recognition-Transduction-Acquisition (RTA) triad.

Examples of Components	Reference
Mobile phone sensing hardware	-
Optical sensing	[47,56,57]
electrochemical sensing	[48,58]
Mobile phone sensing software	-
cloud-based analytics	[69–71]
Drag and drop analytics	[72–78,81–83]

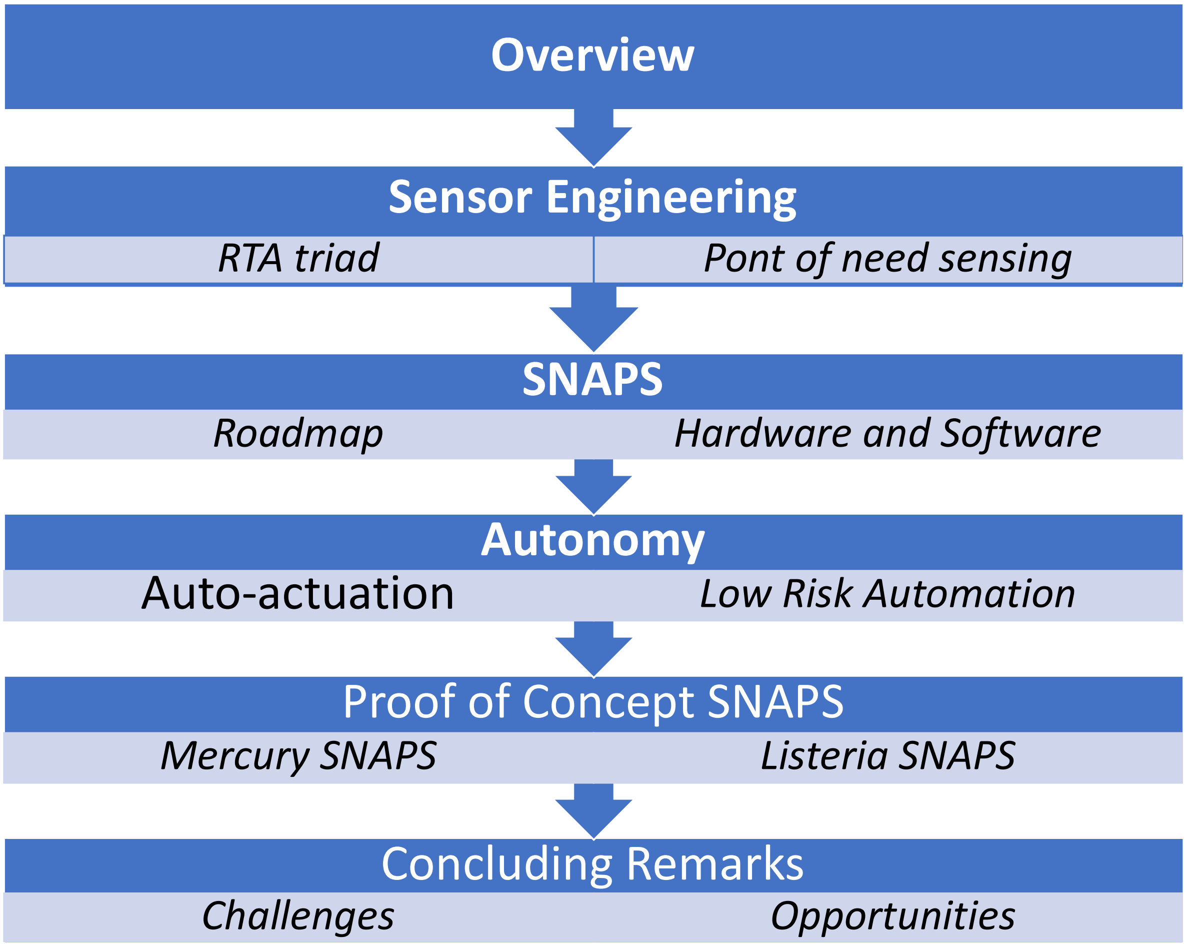

Figure S1. Overview of Review.

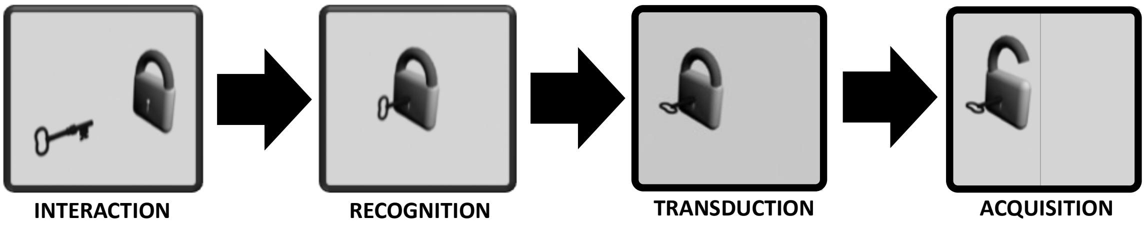

Figure S2. Recognition-Transduction-Acquisition (RTA) triad.

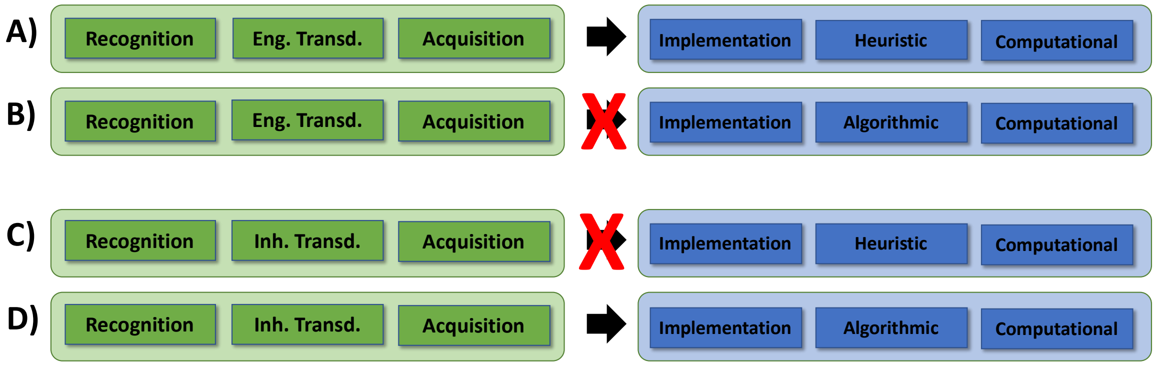

Figure S3. Design of SNAPS must use a retrosynthetic approach, beginning with the intended application in mind. This allows proper selection of materials, transduction techniques, and analytics for ensuring quality of service. (**A**) Correct matching of engineered transduction with heuristic analytics. (**B**) Incorrect matching of engineered transduction with algorithmic analytics leads to overdesign of the tool, consuming unnecessary energy and computational power. (**C**) Incorrect matching of inherent transduction with heuristic analytics leads to excessive data collection, which causes systematic negative effects unless the data. This approach is valid for long term monitoring programs, but is not relevant for rapid, point of need SNAPS. (**D**) Correct matching of engineered transduction with algorithmic analytics.

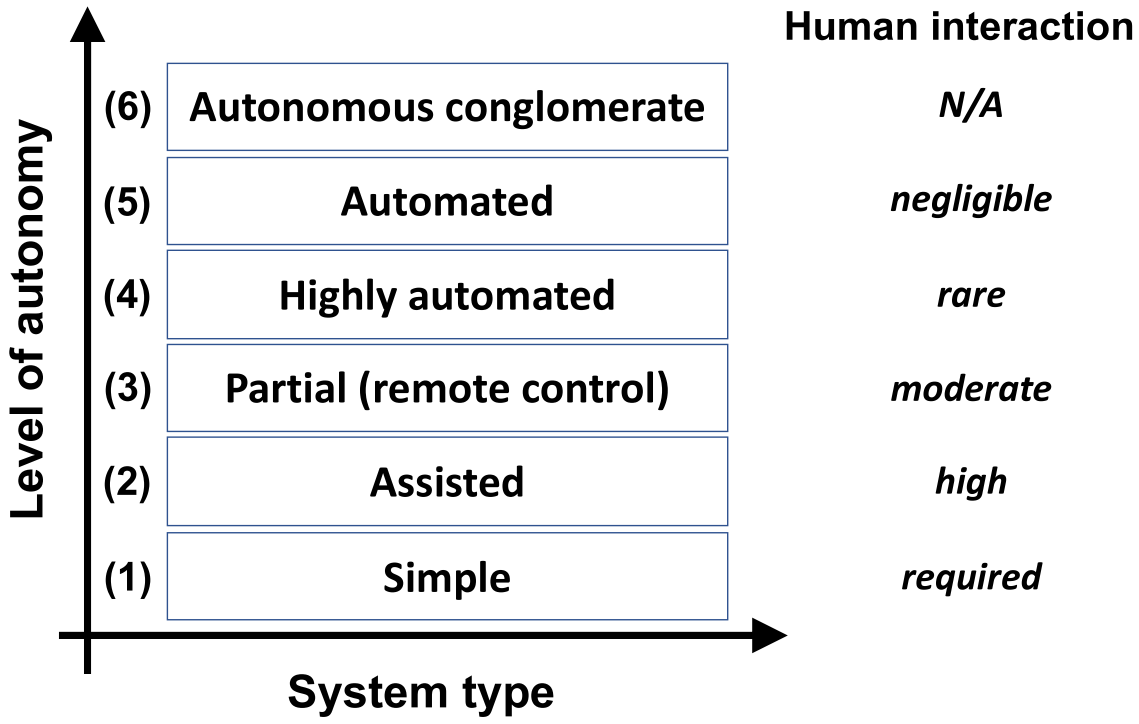

Figure S4. Traditional autonomy may not be the dogma for development of SNAPS.

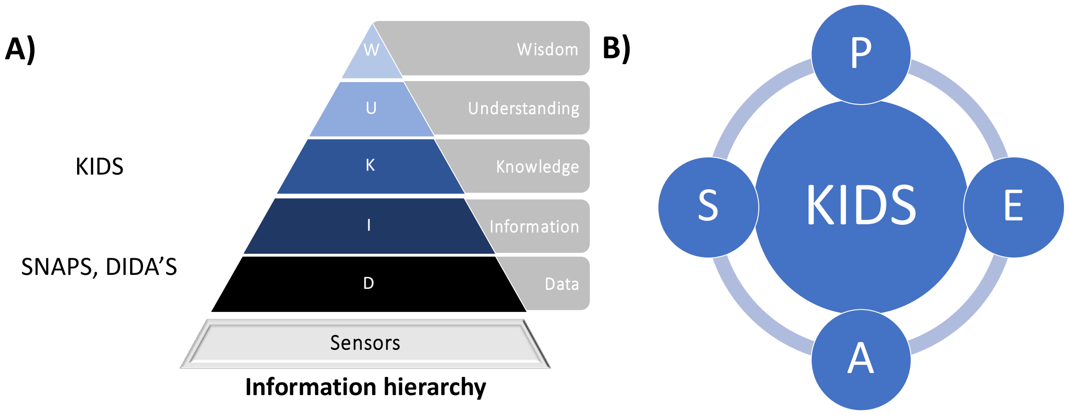

Figure S5. (**A**) Information hierarchy depicting the evolution of sensor data towards knowledge. Higher levels (wisdom, understanding) may be beyond the scope of sensor data, but here we describe a platform for evolution of sensor data to information (SNAPS-ART and SNAPS-DIDA'S) and suggest a path forward for evolution to knowledge via the KIDS platform. (**B**) When KIDS is applied to the agro-ecosystem, the convergence of performance metrics, environment, actuators, and sensors (PEAS) encompass the platform through agent-based systems.

SNAPS-Listeria Monocytogenes

The program below reads raw impedance data files, converts the data to the necessary form for machine learning classification, and predicts whether the tested sample "may be contaminated" or is "safe". When run via R-Studio, the program prompts the user to attach an impedance data file and classifies the results using training data as described in previous publications. This program can be utilized directly (without the app shown in the manuscript) using R Programming Compiler app by Kappsmart.

In the bagged random forest analysis, the number of variables for each split was equal to one third of the total available features (150 in this dataset). The random forest model was generated using the randomForest() function in R. The predict() function from the stats package was utilized to predict the binary response of the "unknown" test sample based on the generated model. The program below may be altered to utilize other classification machine learning techniques such as neural networks and support vector machines for rapid pathogenic contamination determination.

R Code for Mobile Phone Using Compiler App:

```
setwd(dirname(rstudioapi::getActiveDocumentContext()$path))
set.seed(0)
#IMPORT TRAINING DATA
RawData =
read.csv("https://www.dropbox.com/s/b52smaqt618qq4t/TrainingDataSet.csv?raw=1", header =
T)
#Remove Name Column
TrainDataForest = RawData[,c(2:152)]
#Converting binary classification label to a factor
TrainDataForest[["Label"]] = factor(TrainDataForest[["Label"]])
#RANDOM FOREST (Bagged)
library(randomForest)
bagged = randomForest(Label~., data=TrainDataForest, mtry=150, importance=TRUE)
#Open Impedance Data File for the "Unknown" Food Test Sample
library(tcltk)
RawTestSampleData = read.csv(file="stdin",header=T)
#Separates Out Real and Imaginary Impedance
```

```
SampleRealZ = data.frame(RawTestSampleData[,(1:1)]) ; colnames(SampleRealZ) = "Z"
SampleImaginaryZ = data.frame(RawTestSampleData[,c(2:2)]) ; colnames(SampleImaginaryZ) = "Z"
#Combines Impedances into One Column then Transposes The Dataset into the Same Format as the Training Data
SampleTestColumn = rbind(SampleRealZ, SampleImaginaryZ)
SampleTest = data.frame(t(SampleTestColumn))
#Classifies Test Sample Dataset Based on Training Data
SamplePred= predict(bagged, SampleTest, type = "class")
SamplePredValue=as.numeric(paste(SamplePred))
#Displays Results of Classification as Possible Contaminated or Safe
if(SamplePredValue > 0)
{print("Based on data anlysis, the sample is may be CONTAMINATED, please hold for further validation")} else {print("Based on data anlysis, the sample is SAFE!")}
```

R Code for Windows Surface/PC using R-Studio:

```
#Choose Working Directory via Dialogue Box
setwd(choose.dir(default = "", caption = "Select Your Working Directory"))
set.seed(0)
#IMPORT TRAINING DATA
RawData = read.csv("TrainingDataSet.csv", header = T)
#Remove Name Column
TrainDataForest = RawData[,c(2:152)]
#Converting binary classification label to a factor
TrainDataForest[["Label"]] = factor(TrainDataForest[["Label"]])
#RANDOM FOREST (Bagged)
library(randomForest)
bagged = randomForest(Label~., data=TrainDataForest, mtry=150, importance=TRUE)
#Open Impedance Data File for the "Unknown" Food Test Sample
library(tcltk)
RawTestSampleData = read.csv(tk_choose.files(caption = "Select Test Sample Dataset"))
#Separates Out Real and Imaginary Impedance
SampleRealZ = data.frame(RawTestSampleData[,(1:1)]) ; colnames(SampleRealZ) = "Z"
SampleImaginaryZ = data.frame(RawTestSampleData[,c(2:2)]) ; colnames(SampleImaginaryZ) = "Z"
#Combines Impedances into One Column then Transposes The Dataset into the Same Format as the Training Data
SampleTestColumn = rbind(SampleRealZ, SampleImaginaryZ)
SampleTest = data.frame(t(SampleTestColumn))
#Classifies Test Sample Dataset Based on Training Data
SamplePred= predict(bagged, SampleTest, type = "class")
SamplePredValue=as.numeric(paste(SamplePred))
#Displays Results of Classification as Possible Contaminated or Safe
if(SamplePredValue > 0){msgBox <- tkmessageBox(title = "Is this sample safe?",
   message = "Based on data anlysis, the sample may be CONTAMINATED, please hold for further validation.",
   icon = "warning", type = "ok")} else {
      msgBox <- tkmessageBox(title = "Is this sample safe?", message = "Based on data anlysis, the sample is SAFE!",
      icon = "info", type = "ok")}
```
